# Supplementary material for: Patterns of Intron Gain and Loss in Fungi
Source: PLoS Biol. 2004 Nov 30;2(12):e422. doi: 10.1371/journal.pbio.0020422 (PMC532390; doi:10.1371/journal.pbio.0020422)
Supplement: Table S1 — Also available at http://genes.mit.edu/NielsenEtAl/. (4.3 MB ZIP). [file pbio.0020422.st001.zip › NielsenEtAl/html/198.html]

AN7600.1.NCU05238.1.MG04144.1.FG02482.1


```
 CLUSTAL W (1.82) Multiple Sequence Alignments - Introns Inserted


Sequence 1: NCU05238.1	1520 aa
Sequence 2: MG04144.1	1533 aa
Sequence 3: FG02482.1	1535 aa
Sequence 4: AN7600.1	1528 aa
Alignment Length: 1547 aa
Number Identitical Residues: 854 aa
Alignment Score (without introns) 40577


MG04144.1 	-MAAAPGSISTPEEAV1ARIAYISSDVVISVQPSLATETDFSRYLKSAAARKEPSLVAKN
NCU05238.1	--MASTTQIKTPQEAV1ARIAYLASDVVVSVQPSLAAESDFSATLKSLASSRTQSLVAKA
FG02482.1 	MDAATQHQVKTPEEAV1ARIAYLSSDVIVSVQPAIGTDSEFSSHLNRYAGRKDSSLVAQS
AN7600.1  	-----MAPVTSAGEAV1ARIAYQASEVVLSVQPSLQSDSWFTKTLKSLKAAGARSVVPEG
          	        :.:. *** ***** :*:*::****:: ::: *:  *:   .    *:*.: 

MG04144.1 	PNNVPE~IQSVRHNADPLLSVFTPIRSGRFVSVTTTSNILLPSIAHLYKLAEYPVVIHVA
NCU05238.1	ADAVTE~IIPVRHNNDPLLSVFTPIRSGRLVSVTTTSEVLLPSIAHLYKLAQLPVVLHVA
FG02482.1 	ADTVPE0IIPVRHNTDPLLSVYTPTRAGKLVSVTTTSTILLPSVSHLYKLANYPVVLHVS
AN7600.1  	---VPD0VVSVRYNEDPFLSAFHPLQSGKVVSAVTSSSTLLTSIPHLYRLANSPVVIHVA
          	   *.: : .**:* **:**.: * ::*:.**..*:*  **.*:.***:**: ***:**:

MG04144.1 	LHPDRFPDFSSITAIRNSGWTFLQSESIQEAQDMAMTAHALAIRSGKGVIHFYSPWVLSN
NCU05238.1	LGPKSLPDYTAITSIRNSGWTFLQSNSLQEAQDLALTAHALAVQSGKGVIHFFDPSSSAS
FG02482.1 	LQPRTFPDYSVITAIRNSGFTFVQSETLQEAQDLALTAHALAIRTGKGVIHFFAPSTSAN
AN7600.1  	LEPYPFPDYSAISSIRQCGFTFLHSETLQEAQDIALTAHALARKSGKGVIHFFDPANSAN
          	* *  :**:: *::**:.*:**::*:::*****:*:****** ::*******: *   :.

MG04144.1 	DEPVEREGLAVIREILDIDSVRRYQS--APISASGLYADDGRTVLASDQPEQVPSGAASA
NCU05238.1	EQPIAAEDLSLVQEILNLDNVRRFQS--ASISGSSIYANDGRVAVVSEQSEPVAS-----
FG02482.1 	DKPIGVEDASVVRDVLDIESVRRYQAGSAPSSQTGIYADDGHIAVSSDHDAPATTG----
AN7600.1  	DDAIEQEDAQTLKSILDLSRSAVSHS--EGHGVDTLYAETGRVATVSDETVESSTAAQ--
          	:..:  *.   ::.:*::.     ::     .   :**: *: .  *:.    .:.:   

MG04144.1 	PLNGLSSGAISHATSQANSSQVSVKSSENSTSATPHS-TSSATTIEAPPTAVSSDDIYKY
NCU05238.1	-QTVLDARQVEAATATATDSLAPAQDGSDESAQTSQE-QSEASEASATPSASVATTIDEQ
FG02482.1 	AASGNLTVPPSANASKAPSAGTSVKSSQDSESSTPVAPSSVATTVESAPPQVTSEDIYKS
AN7600.1  	VEETATPTQPSQTPSSRSVANSSVGSSERDSSVDSRATSSAATTVDGSVRPVSATDIFSW
          	      .   .  .:    :  .. ... . :  .  . * *:  ...     :  * . 

MG04144.1 	VTSIWAQLESLSGRKYS~PFEYSGPPTPENCLFVFGADAGLLGSAIDEAKSGDVYAKAGI
NCU05238.1	AP------VVTSEHIYN2AFEYSGPSNAENALFVFGSGSPLFAQAIAQAQSDDSFAKAGV
FG02482.1 	AGRIWAHLKTAVGREYS~AFEYSGPPNAENCLFIFGSDAGAFAQAIDEANTQEIFANTAI
AN7600.1  	ASQIWKALSEITGRNYR~AIEYTGPSDAKSAIFVFGS-TGVFVDVLSKEDVPAELTNIGL
          	.            : *  .:**:**. .:..:*:**: :  : ..: : .     :: .:

MG04144.1 	LTPRLYRPWLGAKLLEAIPKSVKKIAVLEQISKRTTKWGPILIDVLTSVKSGPGG--VET
NCU05238.1	LTARLYRPWLGAKLLEAIPKSVKRVAVLEQVSRKTTKWGPLLIDVLTSVKSGPGG--VQT
FG02482.1 	LTPRLYRPWLGSRLVEAIPRSVKRIAVLEQIHRKTTKWGPLLIDVLTSVKSGPGG--VEA
AN7600.1  	ITARLYRPWVGAEIANSIPRSIEKIAVLEQVR-KTTRWGPSFMDLLSSLTPAAVGGRSPQ
          	:*.******:*:.: ::**:*::::*****:  :**:*** ::*:*:*:.... *.    

MG04144.1 	IVGYHVGYINNETVKQALRGICQNLALDKPVQNLEVGSREGPQKADKYALEKPKLETAYT
NCU05238.1	IVGHQLGYISQETVVQALRGIFQNLTSEKPVQNLEVGEREAPKEASEYGLEKPKLETAYT
FG02482.1 	IVGHQLGYIAPEVVVQALRGVLQNLTAEKPIQNLEVGKKEAWKEPTGYDLKAPQLETAYT
AN7600.1  	IVGYRLGYVEPSTAVQALRGIIQNLSSPSPIQSLEIGSSNVP--TLQTPLEQPRIENAYL
          	***:::**:  ... *****: ***:  .*:*.**:*. :    .    *: *::*.** 

MG04144.1 	KILDQLFGTRLYTANAIDSDNAGISATISATPEFGFGALLARKEHRHRFVSEAKEAATSG
NCU05238.1	KILDQLFGERAYIANSIEKDNAGISNTISATPEYGFGALLARKERKQKFVSEVKEAASNG
FG02482.1 	KILDQLFGQRAYVANAIKSSTTGISPTVSASPEFGFGSLLARKQRRGQFVSQVKDAATNG
AN7600.1  	KILNQLFGERLYLANQLSSNNAGISSTIAASPEYGFGSLIARKEHRQRFIREVEAASKSN
          	***:**** * * ** :....:*** *::*:**:***:*:***::: :*: :.: *:...

MG04144.1 	VFLTEKPKRLLTNWAIHSDDPKKIDAAAEDLISELTLENSTAARKLLKNQAFFRRQSLWL
NCU05238.1	QFSSDAPKQALAKWVANAEDSKKVEEVASEAVSKLSEDGSDLSKSLLEYKQFFRKQSLWL
FG02482.1 	SFTTEAPKSWLARWAMNADDASKSTEIADDVIARLETDGSPLAAQLLTAKGLFRKESLWL
AN7600.1  	SFATDVVSKWLADWALNATDATKANRLAPDVIARLSNDGSALSKQLLQTKKLFYSESQWL
          	 * ::  .  *: *. :: *..*    * : ::.*  :.*  : .**  : :*  :* **

MG04144.1 	VGSDAWAYDLGNSGVHHVLASGENVNMLIIDSQPYSEHTAADANKRKKDIGLYAMNFGNA
NCU05238.1	VGSDAWSYDLGNSGVHHVLASGENVNLLIIDSTPYSERAAADANRRKKDIGLYAMNFGNA
FG02482.1 	TGSDAWSYDLGNSGVHHVLASGENVNMLIIDSTPYSERAAADANRRKKDIGLYAMNFGNV
AN7600.1  	IGSDAWAYDLGNSGVHHVLASGADVNMLVIDSQPYSERSAADPTRRKKDIGLYAMNFGNA
          	 *****:*************** :**:*:*** ****::***..:**************.

MG04144.1 	YVASVAAYSSYTQVLQAMDEADKFNGPSIVLAYLPYNGEHDSPLTVLQETKKAVDLGYWP
NCU05238.1	YVASTAVYSSYTQVLQAMDEADKFNGPSVVLAYLPYFGEHDSALTVLQETKKAVDIGYWP
FG02482.1 	YVASTAVYSSYTQVLQALLEADKFEGPSVVLAYLPYFGETDSPLTVLQETKKAVDTGYWP
AN7600.1  	YVASVAVYSSYTQVLQAMAEAEQFKGPSIVVAYLPYNQENDSALTVLQETKKAVDLGYWP
          	****.*.**********: **::*:***:*:*****  * **.************ ****

MG04144.1 	LYRWNPDNEKQGLPTFSLDSERIKKELKAFLDRDNHLTQLMKKEPSFGASLAQDFGTEVR
NCU05238.1	LYRWNPENEKKGEPSFSLDSERIKKELKEFLDRDNHLTQLMKKEPSFGAVLSEDFGTEIR
FG02482.1 	LYRWNPENEKKGEPNFSLDSELIKQELKTFLARDNQLTQLASKEPKFAAALVKDFGSEVR
AN7600.1  	LYRWNPANEDKGEPKFALDSERVKRELEEFLRRDNQLTQLMNRQPKFSSVLSESYGTEVR
          	****** **.:* *.*:**** :*:**: ** ***:**** .::*.*.: * :.:*:*:*

MG04144.1 	AQQKRRARDAYNQLLEGLFGAPLTILFGSDNGNAQSLAKRLGNRGRQRGLKTTVMAMEDY
NCU05238.1	AQQKRKAKDAYNQLLEGLFGAPLTILYASDNGNAISLSKRLGNRGRARGLKTTVMSMEDY
FG02482.1 	AQQKRKAKDAYNQLLEGLLGAPLTVLYASDNGNSTTLAKRLASRGRARGLKTTVLAMEDY
AN7600.1  	AIQKRKAKDSYEKLLEGLFGAPLTILFASDGGNAATLAKRLGNRGRARGLKTLVMAMEDY
          	* ***:*:*:*::*****:*****:*:.**.**: :*:***..*** ***** *::****

MG04144.1 	PVEDLPTEENIVILTSTAGQGEFPQNGKPFWDAIKDSTELDLASVRFSVFALGDSHYWPR
NCU05238.1	PVEDLATEENIVFITSTAGQGEFPQNGLSFWHAVKDNTSLDLANVRYSVFGLGDSHYWPR
FG02482.1 	PLEDLPTEENIVFITSTAGQGEFPQNGLPFWDAIKDNTDLDLAAVNYSIFGLGDSHYWPR
AN7600.1  	PFEDLPTEENVVFITSTAGQGEFPQNGRALWEYVKNTGDLDLSTINYSVFGLGDSHYWPR
          	*.***.****:*::************* .:*. :*:. .***: :.:*:*.*********

MG04144.1 	KEDKIYYNKPGKDLDRVLANFGAQRLAEIGLGDDQDPDGYQTGYAEWEPKIWQALGVDNV
NCU05238.1	KEDKIYYNKPAKDLDRVLANFGAKPLAPIGLGDDQDPDGFQTGYSEWEPKLWEALGVANV
FG02482.1 	KEDKIYYNKPAKDLDRVLTNLGGKHLIDIGLGDDQDPDSFQTGYKEWEPKLWAALGVDKV
AN7600.1  	KEDKIYYNKPAKDLDARIAFLGGRKLTDIGLGDDQDPDAFQTGYSEWEPRIWQALGVDKV
          	**********.****  :: :*.: *  **********.:**** ****::* **** :*

MG04144.1 	EGLPEEPAPITNEDIKLASNYLRGTIVEGLNDNSTGAISAADGQLTKFHGTYMQDDRDVR
NCU05238.1	DGLPEEPAPRTNEDIKIESNYLRGTIVQGLNDTSTLAISPDDCQLTKFHGTYMQDDRDIR
FG02482.1 	DGLPDEPPPITNEDIKVASNYLRGTIVEGLNDTSTLAISAADQQLTKFHGTYMQDDRDIR
AN7600.1  	EGLPEEPAPLTNEDIKIQSNYLRGTIAEGLLDESTGAISASDQQLTKFHGTYMQDDRDVR
          	:***:**.* ******: ********.:** * ** ***. * ***************:*

MG04144.1 	DERKAQGLEPAYSFMIRCRLPGGVSTPQQWVQMDDIATKLGNETMKLTTRQTFQFHGVVK
NCU05238.1	DERKAQGLEPAYSFMIRCRLPGGVSTAKQWVQMDDIANELGNETMKLTTRQTFQFHGVVK
FG02482.1 	DERKAQGLEPAYSFMIRCRLPGGISTPQQWVQMDDIANELGNETMKLTTRQTFQFHGVVK
AN7600.1  	DERKAQGLEPAYSFMIRCRLPGGVATPSQWLQMDAISSAYGNETMKLTTRQTFQFHGVVK
          	***********************::*..**:*** *:.  ********************

MG04144.1 	GKLKPAMQAINRALMTTIAACGDVNRNVMCSSLPTQSEYHREVYACSQKISDHLLPSTTA
NCU05238.1	SKLKPAMQAINRALMTTIAACGDVNRNVMCSPLPAHSKYHRAVHACAKRISDHLLPNTTA
FG02482.1 	SKLKPAMQAINRALMTTIAACGDVNRNVMCSSLPTQSKYHREVFACSQLISDHLLPSTSA
AN7600.1  	RNLRGAMRAINKALMTTIAACGDVNRNVMCSSLPELSYYHREVHAVSKKISDHLLPATTA
          	 :*: **:***:*******************.**  * *** *.* :: ******* *:*

MG04144.1 	YHEIWLTDDNDKKTMVAGDAVQDFEPLYGPTYLPRKFKITIAIPPHNDTDVYAHDIGLIA
NCU05238.1	YHEIWLTDDDGAKTQVGGNAVQDFEPLYGPTYLPRKFKVSIAIPPHNDVDVYAHDIGLIA
FG02482.1 	YHEIWLTDDDNKKTQVAGDAVQDFEPLYGPTYLPRKFKITIAIPPHNDTDVYAHDIGLIA
AN7600.1  	YHEIWLQDENDKKVQVAGDAVVDQEPLYGPTYLPRKFKITIAIPPHNDTDVYAHDIGLIA
          	****** *::. *. *.*:** * **************::********.***********

MG04144.1 	IKGKDGHLEGFNLLAGGGMGATHNNKKTYPQTGRSFGYVSKDQVHIACEKVMLVQRDHGD
NCU05238.1	IKNEDGSLAGFNLLAGGGMGATHNNKKTYPQTGRMLGFVTAADVHLACEKVMLVQRDNGD
FG02482.1 	IKGEDGKLAGFNVLAGGGMGATHNNKKTYPQTGRMFGFCKTEDVHIVCEKIMLVQRDNGD
AN7600.1  	IKGADGHLEGFNILAGGGMGTTHNNKKTYPQTGRMFGYVPADQAHIVCEKIMLVQRDYGD
          	**. ** * ***:*******:************* :*:    :.*:.***:****** **

MG04144.1 	RKNRKHARLKYTIDDMGVDVFRGKVEELW--GQKFEEARPFHFDSNIDTFGWQRDEKGLN
NCU05238.1	RKNRKHARLKYTIDDMGVDVFKAKVEEYW--GQKFEEPRAFHFDSNVDTFGWTKDETGLN
FG02482.1 	RKNRKHARLKYTIDDMTVPVFRSKVEELW--GKKFENERPFEFKSNVDTFGWQKDEHGLN
AN7600.1  	RKNRKHARLKYTIDDMGVEAYKEKVEALLPDGLRFAEPRPFKFASNVDTFGWQKDENGLN
          	**************** * .:: ***   ..* :* : *.*.* **:***** :** ***

MG04144.1 	HFTLFIENGRIEDTAEFQMKTGLREIAKVHKG--EFRLTGNQHVILSNVADEDLDNMKAL
NCU05238.1	HFTMFIENGRIEDTAEFQMKTGLREIAKVHKG--EFRLTPNQHLILSNVADEDKPELEKL
FG02482.1 	HFTFFIENGRIEDTPEFQMKTGLREIAKSLKGESEFRLTGNQHLILSNVADEHLDDVKEL
AN7600.1  	HFTFFIENGRIEDTADFRMRTGLRELASLDKG--EFRLTGNQHLILSNIKDEDLPAIKEL
          	***:**********.:*:*:*****:*.  **  ***** ***:****: **.   :: *

MG04144.1 	LKKYKLDNIQFSGLRLSSSACVAFPTCGLAMAESERYLPVLIDKLEACLEENGLKQDSIV
NCU05238.1	LAQYKLDNLHHSGLRLSSSACVAFPTCGLAMAESERYLPVLITKLEGVLEEVGLKQDSIV
FG02482.1 	MKKYKLDNVQFSALRLSSSACVAFPTCGLAMAESERYLPVLISKLEGCLEENGLRQDSIV
AN7600.1  	MAKYKLDNTSFSGLRLSSSACVAFPTCGLAMAESERYLPVLITKLESTLEEVGLARDSIV
          	: :*****  .*.***************************** ***. *** ** :****

MG04144.1 	MRMTGCPNGCARPWLAEVAFVGKAYGAYNMYLGGGYHGQRLNKLYRSSIKEDEILAIMKP
NCU05238.1	MRMTGCPNGCARPWLAEVAFVGKAYGAYNMYLGGGYHGQRLNKLYRSSIKEEEILTIMKG
FG02482.1 	MRMTGCPNGCARPWLAEVAFVGKAYGAYNMYLGGGYHGQRLNKLYRQSIKEDEILDIMKP
AN7600.1  	MRMTGCPNGCARPWLAEVAFVGKAYGAYNMYLGGGYHGQRLNKLYRSSIKEDEILDIMKP
          	**********************************************.****:*** *** 

MG04144.1 	LLKRYALER----EEGERFGDFCIRAGVIKATTDGQNFHND0VAEEESDEE
NCU05238.1	LLRRYAKER----EEGERFGDWTIRAGIIKATTDGRNFHEG0VAEEEGDEE
FG02482.1 	LLKRYALER----NEGERFGDFCVRSGVIVATTDGQNFHDN0VADEEEDDE
AN7600.1  	LLKRYALERNTDGEEPERFGDWLIRAGVIKETTDGRNFHEG0VAEEEDEE-
          	**:*** **.:..:* *****: :*:*:*  ****:***:. **:** ::
```
